# Color stability of nanohybrid and microhybrid composites after immersion in common coloring beverages at different times: a laboratory study

**DOI:** 10.1038/s41405-023-00161-9

**Published:** 2023-08-16

**Authors:** Ahlam Mohammad Al-Shami, Mohammad Ali Alshami, Abdulwahab I. Al-Kholani, Amat-Alkhaliq Mohammad Al-Sayaghi

**Affiliations:** 1 Department of Conservative Dentistry, Faculty of Dentistry, Sana’a University, Sana’a, Yemen; 2 Department of Dermatology and Venerology, Faculty of Medicine and Medical Sciences, Sana’a University, Sana’a, Yemen

**Keywords:** Bonded restorations, Oral cancer detection, Periodontitis, Dental pulp, Peri-implantitis

## Abstract

**Objective/Aim:**

This in vitro study aimed to evaluate the color stability of microhybrid and nanohybrid restorative composites after exposure to immersion media common in Yemen for different periods.

**Materials and methods:**

Two composite materials, nanohybrid Tetric N-Ceram and microhybrid Te-Econom Plus, were investigated. Six groups of 30 cylindrical specimens (*n* = 5/group; diameter, 10 mm; thickness, 2 mm; shade A2) of each restorative material were immersed for 1 week in distilled water, qat solution, Yemeni coffee, traditional Yemeni coffee (qishr), red tea, and Dilsi cola. Color changes were evaluated by colorimetry. The color data and pH were measured before and 1, 3, and 7 days after immersion. The data were statistically analyzed.

**Results:**

Tetric N-Ceram showed lesser discoloration than did Te-Econom Plus. Qat, coffee, and red tea caused highly significant discoloration than did Dilsi cola and distilled water (*p* < 0.05). The role of low pH in discoloration depended on the colorant.

**Discussion:**

Nanohybrid Tetric N-Ceram composites are more resistant to discoloration than are microhybrid Te-Econom Plus composites. Qat and coffee have the highest effect on composite discoloration.

**Conclusions:**

These findings will aid in selecting composite materials and patient instruction.

## Introduction

Developments in conservative restorative materials and adhesive systems have revolutionized dental practice. The possibility of bonding the restorative material to the dental surface and innovations in esthetic procedures have enabled more conservative processes in tooth preparation. The outcome of a restoration is positively correlated with the quality of the filling material [1]. Resin composites are mostly used in conservative dental restorations instead of amalgam owing to their excellent cosmetic properties and ability to bond strongly to the tooth. Revision or replacement of composite restorations, which is costly and time-consuming, is commonly required because of discoloration [2]. Both the science and technology of composite dental restorative materials have advanced considerably, resulting in the development of new composite resin materials [3]. Composite dental materials have been designed to increase longevity and color stability after placement in the oral cavity [4]. Manufacturers have introduced different composite resins to achieve the following requirements: light-curability, perfect color match, and stability [5]. These resin materials include condensable/packable, flowable, microhybrid, and nanocomposite materials [6].

Discoloration, the most common reason for composite restoration replacement, is generally caused by dietary products, such as coffee, tea, and nicotine [7]. Restorative material discoloration might be attributable to matrix resin hydrophilicity and the degree of water absorption. If a composite resin can absorb water, it can also absorb other fluids, resulting in color alteration [8]. Filler particle size and distribution, as well as resin matrix composition, play an important role in this context [9]. The photoinitiator system can influence the polymerization characteristics and affect the color stability of the composite [10].

Many factors affect the color stability of resin composite restoration materials; these include patient habits, such as beverage consumption, smoking, or qat chewing, as well as colorants that may be incorporated into food and drinks. These staining media lead to the absorption of pigments, which affects the color of the material. Many factors affect the speed of the degradation reaction, including pH, matrix, type of chemical bond filler, water uptake, and copolymer composition. In particular, low pH is an undesirable factor for hydrophilic resins owing to its impact on degradation rates during catalysis.

Qat chewing is a widespread habit among the Yemeni population [11]. The leaves and tender small twigs of qat (*Catha edulis*) are put inside the cheek and chewed for more than 4 h for amphetamine-like effects [12]. Qat has been in use for centuries in many countries, especially in the Middle East and east Africa. The discoloration of teeth and dental materials may occur due to the chronic use of qat [13].

Traditional Yemeni ginger coffee (qishr) is a sugary-tasting beverage made from the husks of coffee beans. This type of hot drink is slightly lighter than tea in flavor and color. People drink qishr at any time of the day [14]. In addition, no study or data available in Yemen has yet evaluated the effect of most beverages commonly consumed by the Yemeni population.

Therefore, this study aimed to assess the effect of staining beverages, especially the most commonly consumed Yemeni drinks, and qat on the discoloration resistance of Tetric N-Ceram and Te-Econom Plus composites materials, which are widely used by Yemeni dentists. The color changes of microhybrid and nanohybrid restorative materials and the pH of the immersion solutions were evaluated for different staining agents for time periods of up to 1 week. The null hypothesis was that the beverage type, period of immersion, and pH will not affect the color stability of various composite resins.

## Materials and methods

This laboratory-based study was conducted to determine the color stability of a new generation of direct restorative composite resins. The nanohybrid (Tetric N-Ceram) and microhybrid (Te-Econom Plus) composite resin restorative materials utilized in the study, along with their manufacturers and chemical compositions, are presented in Table 1.

**Table 1:** Composite materials used in this study.

Material	Product	Category	Composition	Manufacture	Shade
Resin-based composite	Te-Econom plus	M	Consists of: dimethacrylate and TEGDMA(22 wt.%). The fillers include barium glass, ytterbium tifluoride, silicon dioxide, and mixed oxide (76 wt.% or 60 vol.%). Additives, initiators, stabilizers, and pigments are additional contents (2 wt.%). The particle size of inorganic fillers is 0.04–7 nm. The mean particle size is 850 nm	Ivoclar vivadent clinical AG Schaan/Liechtenstein	A2
Resin-based composite	Tetric N-ceram	N	Consists of dimethacrylates (19–20 wt.%). The fillers contain barium glass, ytterbium trifluoride, mixed oxide and copolymers (80–81 wt.%). Additives, initiators, stabilizers, and pigments are additional content (< 1 wt.%). The total content of inorganic fillers is 55–57 vol.%. The particle size of inorganic fillers is 40–3000 nm.	Ivoclar vivadent clinical AG Schaan/Liechtenstein	A2

M (microhybrid composite) and N (nanohybrid composite) are the two types of composite material (shade A2) used in this in vitro study, which are widely used by the Yemeni population.

Six immersion media were assessed in this study: qat solution, Yemeni coffee, qishr, red tea, Dilsi cola, and distilled water. The pH of the immersion media was verified using a pH meter (HQ411D pH/mV/RedOx/ORP meter, Hach, Loveland, CO, USA), and the color shade was assessed using a colorimeter (Portable Color Difference Meter TCD100, PCE Instruments, Hampshire, UK) before and 1, 3, and 7 days after immersion according to the CIELAB color space system.

### Specimen preparation

**Fig. 1:**
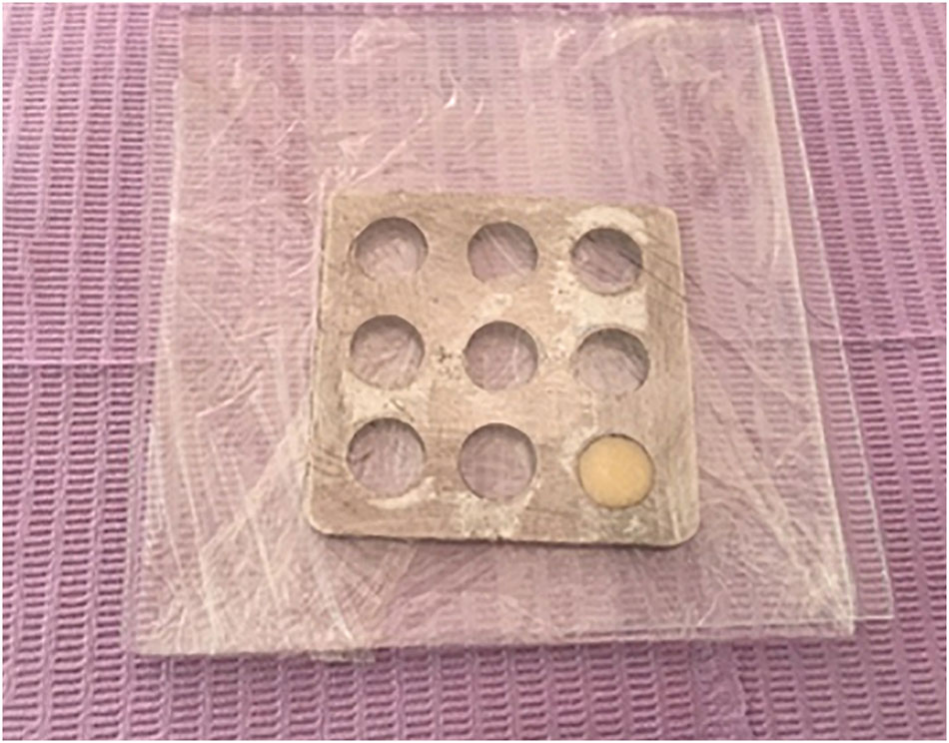
Composite specimen preperation in a metalic mold between two glass slides and a nylon film. Each composite specimen was prepared separately in a metallic mold covered with a nylon film and held between two glass slides.

Sixty composite disc-shaped specimens (30 each of the nanohybrid Tetric N-Ceram and microhybrid Te-Econom Plus) were prepared using prefabricated metallic molds [15–17]. To standardize the sample thickness and diameter (10 mm diameter and 2 mm thickness), a manual caliper was used to ensure accuracy; any sample with a nonconformity, such as surface irregularities, visible cracks, or porosities, was excluded, and replaced with a new sample with accurate specifications. After the insertion of the composite material into the metallic mold, the material was held between two glass slides. Each glass slide was 1.5 mm thick and covered with a transparent nylon strip to form a smooth glossy surface and to avoid oxygen inhibition (preventing the formation of an unpolymerized air-inhibited layer; Fig. 1) [18–20], and the two slides were pressed gently together by compression with a 500 g device for 20 s to allow the escape and removal of excess material [21].

**Fig. 2:**
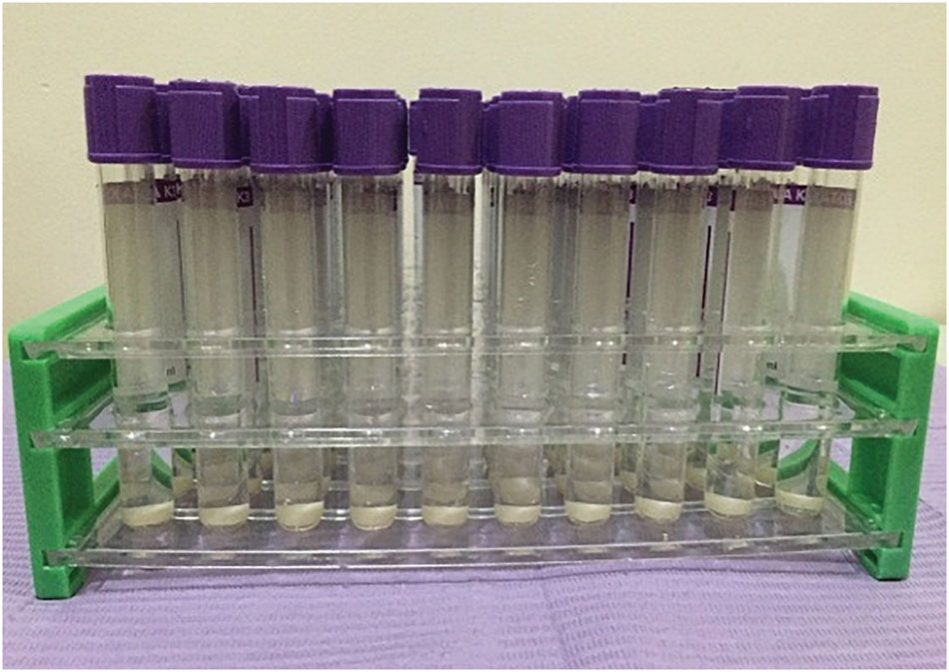
Closed tubes containing specimens stored in distilled water. Composite specimens were stored in closed glass tubes with rubber stoppers and concave bottoms containing distilled water for 24 h before immersion in staining media. The specimens were kept inside the plastic tube holders.

The composite resins were light-polymerized with a light-emitting diode (LED) curing light (MaxCure 9, Guilin Refine Medical Instrument Ltd., China) according to the manufacturer’s instructions (light intensity with an output power of about 1000–2500 mW/cm^2^) with a TURBO mode Display P1 with light intensity 2300–2500 mW/cm^2^. A 1.5 mm-thick glass slide was placed between the tip of the light-curing unit and the composite disc; this maintained the tip of the light-curing unit at a fixed distance of 1.5 mm from the surface of the specimen [22]. After 10 polymerizations, the efficacy of the light-curing device was measured using a dental radiometer (LED light meter, LM-1, Guilin, China). All specimens were removed from the mold, and each surface was further light cured for 1 s to ensure adequate curing [15]. All specimens were polished using a brush, cup, and Polidont kit containing polyester film with aluminum oxide discs (medium, fine, and super-fine) with paste (abrasive discs; Microdont, Brazil) [23], which were used for 10 s using a slow-speed handpiece following the manufacturer’s instructions [16]. The samples were then stored inside closed tubes in distilled water (Fig. 2) and incubated for 24 h at 37 °C for rehydration and polymerization completion [24]. It was expected that polishing helped in creating conditions that were closer to the clinical circumstances [20]. Polishing procedures were performed for 10 s for each step to avoid micro-crack formation [21]. All specimen preparation and finishing procedures were carried out by the same operator to reduce variability.

The samples were divided into six groups, each containing five specimens of each material. Each specimen was stored in a tube to be immersed in different staining solutions (qat solution, Yemeni coffee, qishr, red tea, Dilsi cola, and distilled water). All samples were kept at 37 °C for 24 h using an incubator.

### Immersion media preparation

**Fig. 3:**
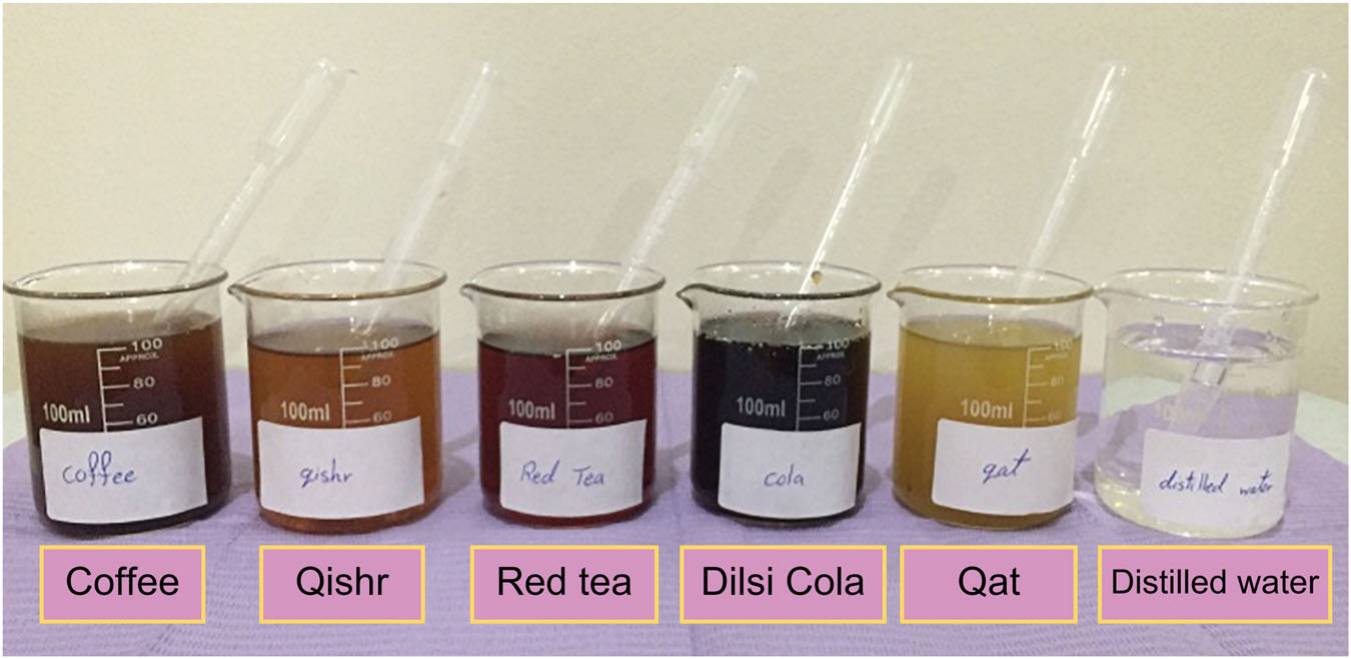
The immersion media. The six immersion solutions used in our study in glass cups with plastic droppers from left to right: coffee, Qishr, red tea, Dilsi Cola, qat, and distilled water.

The immersion media are shown in Fig. 3. The tea solution (Al-Kbous, Sana’a, Yemen) was prepared by immersing two prefabricated tea bags (2 × 2 g) into 300 mL of boiling mineral water free of sugar or milk [25]. The Yemeni coffee solution (Al-Kbous, Sana’a, Yemen) was prepared by immersing 2 g coffee in 100 mL boiling water without milk or sugar [26], and the qishr was prepared by adding 2 g qishr (husks of coffee seeds) in 100 mL boiling water for 10 min without any additional flavor such as ginger or cinnamon; this is the most common way of preparing qishr in Yemen. Dilsi cola (Derhim Industrial Ltd., Yemen) and distilled water (Pure nature-Sana'a Yemen plastic container) were used as received, 100 mL each.

Qat (qatal arhabi qat, Arhab, Sana’a, Yemen) was used for qat extract preparation. Initially, the fresh leaves and twigs were cleaned by washing with water, followed by air drying and mincing. The qat extract was prepared by immersing 20 g of the minced dried material into 500 mL of mineral water (Shamlan, Sana’a, Yemen). It was then stirred at 200 rpm for 5 h at 37 °C. After 5 h of stirring, medium-grade filter papers were used to filter 4 mL of the extract for pH measurement. Subsequently, fresh stock solutions were ready for use [27]. The same procedures were followed daily to obtain a fresh solution of qat, and this method mimics the reality of chewing qat.

The solutions were stirred three times a day [28] by an orbital shaker (TS-1000, Shanghai, China) at a speed of 30 rpm to prevent air entrapment around the specimens, settling, or sedimentation of the solutions [29]. Colorant solutions were renewed every day to avoid bacterial or yeast contamination. The container lids were tightly closed to prevent escape of carbonic gas and maintain an appropriate carbonation level [16].

### Color assessment

The color of the specimens was assessed at four-time points: before and 1, 3, and 7 days after immersion in the media. After sample preparation and immersion in distilled water, color measurements were taken for each specimen using a colorimeter. During color measurement, a white background was used as a baseline. The colorimeter measured the specimen color based on the CIELAB L*a*b* color space system, which allows three-dimensional determination of color. The color differences (ΔE) among the color coordinates were calculated by applying the formula to compare values before and after immersion: ΔE = [(ΔL*)^2^ + (Δa*)^2^ + (Δb*)^2^]^1/2^, where ΔE represents the total color difference and ΔL*, Δa*, and Δb* represent the changes in lightness, red-green coordinates, and yellow-blue coordinates, respectively.

After 24 h, 3 days, and 7 days, color measurements were performed for two different positions of the specimen, and the average was calculated for each specimen.

### Pilot study

To be familiar with the color measurement, immersion solutions, and colorimeter device, a pilot experiment was performed using six specimens of each composite type before the actual experiment.

### Statistical analysis

Data were collected and analyzed using the Excel computer software package (Excel 19; Microsoft) and Statistical Package for the Social Sciences (SPSS, Version 25, SPSS Institute). The Shapiro–Wilk test was used to analyze normality, followed by paired sample t-test and three-way analysis of variance (ANOVA). One-way ANOVA with Scheffé test was applied to analyze statistically significant differences in the test samples and multiple comparisons for adjusting the level of significance to decrease the error rates. The significance level was set at *p* < 0.05.

### Permission

Qat is a cultural plant in many countries including Yemen. Its use is not illegal, and no permission is needed to collect or consume it. and it is available to everyone in many markets across the country.

## Results

All measurement data (a*, b*, L*, and pH of each composite group) were normally distributed according to the Shapiro–Wilk test (*p* > 0.05). A paired sample *t*-test was used to evaluate the reliability, and the value was 0.97, indicating high internal consistency.

We calculated the means and standard deviations of color differences for all staining media at 1, 3, and 7 days, compared with baseline values, for the two composites (ΔL, Δa, Δb). The Te-Econom Plus surfaces exhibited brownish discoloration, with the highest difference after immersion in red tea for 3 days, followed by coffee, Dilsi cola, and qishr to the same degree. Qat caused the least change in the a* axis. The most brownish discoloration of Tetric N-Ceram surfaces appeared after immersion in red tea and coffee for 3 days, followed by qishr, Dilsi cola, water, and qat. The brightness of Te-Econom Plus specimens, represented by L*, was most reduced by distilled water. A yellow-to-green (greenish) discoloration appeared on the surface of all Te-Econom Plus specimens; the most yellow-to-green discoloration was due to qat, followed by coffee, red tea, and qishr; Dilsi cola and distilled water resulted in the lowest. The color changes increased with time, but Tetric N-Ceram specimens showed the most yellow-to-green discoloration after immersion in the decreasing order: Qat>coffee>qishr>red tea>Delsi>distilled water. The color changes increased with increasing immersion time. In general, changes on the b* axis started after 24 h and increased after 3 days, reaching the highest point after 7 days. Changes on the a* and b* axes were clearer than those on the L* axis due to changes in color. In addition, compared with immersion in other staining solutions, immersion in qat resulted in the highest differences for both tested composites.

We performed pairwise comparison of ΔE values at all measured times between all staining media for the two composites. Only the qat solution, coffee, and red tea produced color changes that were clinically perceptible (ΔE > 3.3). Among all different immersion media, the qat solution induced the highest discoloration level. The least discoloration was found with distilled water. Generally, Te-Econom Plus showed more color change than did Tetric N-Ceram. As confirmed by three-way ANOVA, a significant interaction between the two resin composites, six staining media, and different immersion times was observed (*p* < 0.05).

**Fig. 4:**
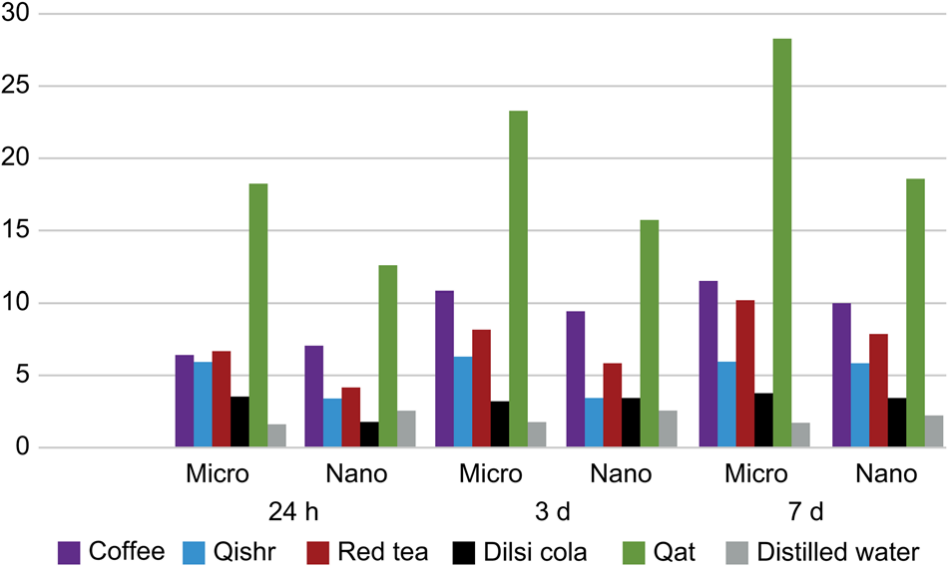
The mean color changes (∆E*) of the different experimental groups over time. The mean color changes (∆E*) of the two types of micro and nano composites, indicated by the height of the vertical column, increasing proportionally with immersion times (1, 3, and 7 days) in different media. It is visible that qat (green column) had the highest discoloration on both types of composites.

**Fig. 5:**
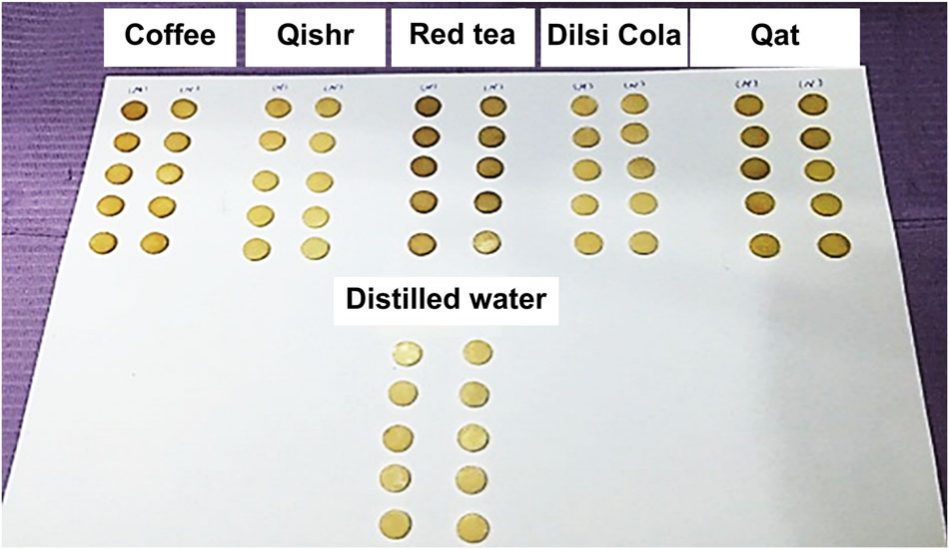
Specimens after 168 h of immersion. Specimens after 168 h of immersion in each type of immersion media had two rows. The first from the left is micro and the second is nano. The highest value was for qat and red tea.

The differences in the ΔE values after staining for both composites (multiple comparisons of ΔE) from the highest to the lowest were as follows: qat>coffee>red tea>qishr>Dilsi cola>distilled water (Fig. 4). Significant differences between Te-Econom Plus and Tetric N-Ceram (*p* < 0.05) were found when the immersion time and staining media were fixed (Table 2). No significant differences were found between 3 and 7 days (*p* > 0.05) in either composite when the material and staining media were fixed. The b* value was the most affected factor in discoloration. All immersion media had acidic pH; Dilsi cola and qat solution were the most acidic. With the increase in immersion time, the solutions became more acidic and discoloration increased; however, the effect of pH indirectly depends on the colorant. The change in color after 168 h of immersion was clear (Fig. 5).

**Table 2:** Multiple comparisons of ΔE values between the two composites.

Dependent variable	Composite		Mean difference	*P*-value
L	Te-Econom plus	Tetric N-Ceram	−0.384	0.027
a	Te-Econom plus	Tetric N-Ceram	0.353^a^	0
b	Te-Econom plus	Tetric N-Ceram	1.287^a^	0

Dependent variable: color change.

For one-way ANOVA, *P* < 0.05; therefore. Scheffé test was applied for multiple comparisons, ^a^difference was applied in dependent variable axis a and b for both materials.

We observed that only qat and qishr media produced gases with foam. When opening the closed tube before 24 h was complete, an acidulous smell was noticed for these two media, more than that for Dilsi cola, which is highly carbonated.

**Fig. 6:**
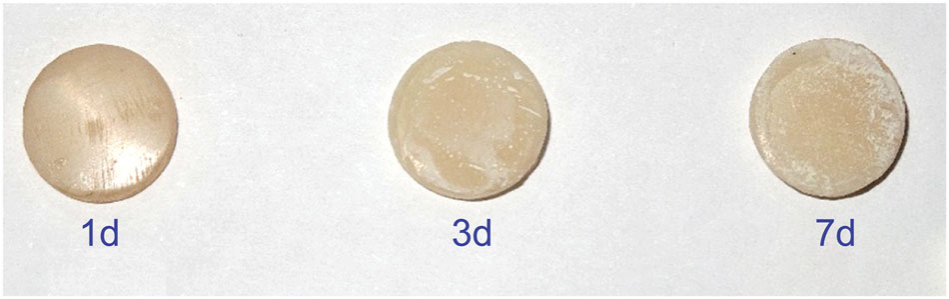
The surface roughness of the samples after immersion in Dilsi Cola. The surface roughness of the samples increased after immersion in Dilsi Cola. The effect of the acidity of Dilsi Cola on the surface of the samples proportional with the time of immersion (1, 3, and 7 days).

Both composite types had physical variations rather than discoloration when immersed in Dilsi cola, and instances of surface roughness were observed (Fig. 6).

## Discussion

Perceptible color changes may compromise the clinical acceptability of a composite restoration [22]. In the present study, the color stability of two conservative restorative materials upon exposure to different staining solutions common in Yemen was evaluated, and the pH was also assessed.

Color changes of both restorative materials depended upon the type of the restorative material, time of immersion, and type of pigmenting media. This result is in agreement with those of Nuaimi and Ragab [25], Özdaş et al. [16] and Choi et al. [30] on the color stability of composite resin restorative materials, which have suggested that the color stability of these resins is unpredictable over a long period of time and affected by the composite type and storage time, with most of the resins showing unacceptable color changes over time. Maranhão et al. [31] suggested that the composition of the composite restoration, type of pigmenting solution, and immersion time determine the color change.

In this study, both resin materials were discolored, but the severity of discoloration depended on the type of immersion media. This finding is in accordance with those of Gupta et al. [32] which suggested that the color match of esthetic restorations in the oral cavity is affected by dietary habits.

Another finding in this study is that the nanohybrid composite was more resistant to pigmentation than was the microhybrid composite. We conclude that this is due to the size of particles, although many factors affect the color stability of resin composites, including extrinsic and intrinsic factors. The extrinsic factors include patient habits of consuming beverages and food containing colorants that lead to the absorption of pigments and affect the color of the material [33].

Intrinsic factors of the composite also affect color stability. The staining ability of the composite is related to the resin matrix, percentage of filler particles, adsorption and absorption mechanisms of stains, and chemical interactions between composites and the stains. The composition of the resin materials and the relative amount of resin and filler content presence influence the color change of a resin material. Resin materials that have lower filler content and higher resin content tend to absorb more water at the resin–filler interface, leading to hydrolytic degradation of the filler. Thus, resin-based materials with lower filler content have poor color stability [34]. Therefore, a composite with large filler particles has more color permeability than does a composite with small filler particles; this explains our result showing that Tetric N-Ceram is more stain-resistant than is Te-Econom Plus.

Additionally, water sorption is mostly due to direct absorption in the resin matrix. Glass filler particles cannot absorb water, yet they can contribute to water adsorption at the surface of the material. The level of water sorption is a function of the resin content of the material and the strength of the resin–filler interface. Excessive water sorption causes plasticization and expansion of the resin, which leads to reduced hydrolysis of saline and longevity of the composite resin, which in turn creates micro-cracks. Thus, the interfacial gaps or micro-cracks at the interface between the matrix and filler allow penetration of staining materials and discoloration [25].

Qat caused the highest color changes in both materials in all time periods in this study; the mean ΔE ranged between 18.1 and 28.0. This finding is in agreement with the results of Al-Anesi et al. [35] which investigated the color stability of highly filled direct and indirect composite materials used in anterior cosmetic restoration. In their study, qat solution was found to cause severe discoloration, particularly at the site of qat inside the oral cavity, and the discoloration was dependent on time. Many factors associated with qat chewing may influence the composite restoration discoloration, such as drinking cola, energy drinks, grape juice, or beverages with ginger or sugar cubes while chewing qat.

The increased discoloration was associated with increasing immersion time. Small values of color changes were observed in the first week, which increased; this explains why most qat chewers who use qat for long periods every day have more discolored teeth than do those who chew only occasionally.

The discoloration by staining media affects the teeth without the loss of enamel luster. Stains associated with teeth are caused by the presence of chromophores (colored agents). Chromophores arise from two chemical sources: organic compounds (i.e., carotene) and inorganic transition metal ions (i.e., iron and tin) [13]. In the case of Yemeni coffee immersion, the observed mean color change (ΔE) values (yellowish) were between 6.4 and 11.4. This result is in accordance with those of Maranhão et al. [31] and Raja et al. [17]. Coffee contains components called tannins, which are a type of polyphenol that breaks down in the water. This component is also found in other beverages, including tea and wine. Tannins are a subgroup of phenolic compounds that cause an unwanted yellowish discoloration on teeth. It only takes one cup of coffee a day to cause staining [22].

Red tea was shown to cause the third-largest color change in both materials in all time periods; the mean ΔE ranged between 6.6 and 10.2. Red tea came after coffee in discoloration, and this finding is in agreement with those of Gupta et al. [32] and Al-Anesi et al. [35]. They reported that tea revealed significantly more color change than did cola and distilled water for all tested composites. They investigated the color stability of highly-filled direct and indirect composite materials used in anterior cosmetic restoration. Tannic acid and staining agents in tea cause significant composite resin color changes [28]. Although the acidity of Dilsi cola might damage the restoration surface, it has a low potential to stain the restoration [15].

The mechanism behind the color alteration of composite restorations after immersion in various liquids has been the subject of great interest in many studies. The absorption and adsorption of colorants with different polarities found in beverages, such as coffee and tea, on the surface of composite restorations have been implicated [36].

With tea and coffee immersion for 30 d, Nuaimi and Ragab [32] also found three nanohybrid composites (Venus Diamond, Ceram X, and Filtek 350 XT) to be more color-stable than are materials with different chemistry, such as the composite-based resin microhybrid Te-Econom Plus. Reddy et al. [37] demonstrated that nanohybrid composites were more color stable than were the other materials tested following immersion in tea, coffee, and cola staining solutions for 1–15 and 30 days. Ertas et al. [3] found that nanohybrid-based materials (Filtek Supreme and Grandio) were more color stable than were three microhybrid composite resins (Filtek P60, Filtek Z250, and Quadrant LC) when immersed in coffee, tea, cola, and red wine for 24 h in a study to determine the effects of different drinks on stainability of resin composites.

The results of our study showed that the effects of coffee, tea, and Dilsi cola decreased in that order. This is inconsistent with the findings of Gupta et al., [32] who noted that the immersion media with the greatest effect on composites is cola, then tea. Surface roughness was observed in both composite samples immersed in Dilsi cola, which is a change in its surface properties rather than discoloration, as observed by Gupta et al. [32] Kumar et al. [15] and Kumari et al. [38]. These researchers concluded that composite resins have surface roughness, abrasion, and degradation when immersed in Pepsi cola. This result explains the discoloration of teeth in qat chewers who also drink Dilsi cola or those who drink Dilsi cola with food or any other beverage with a strong colorant.

The results of this study prove that both Tetric N-Ceram and Te-Econom Plus underwent discoloration after immersion in staining solutions (qat, coffee, qishr, red tea, and Dilsi cola). The microhybrid was more discolored than was the nanohybrid; this result contradicts the results reported by Nuaimi and Ragab [25], who concluded that there were statistically significant differences between Filtek 350 XT and Ceram X or Venus Diamond in tea and coffee storage; however, there were no statistically significant differences in Ceram X or Venus Diamond when immersed in tea and coffee.

Time is one of the multiple factors that affect the discoloration of composite restorations. For Tetric N-Ceram and Te-Econom Plus, time played an important role in discoloration, and the color became more saturated, with discoloration increasing with increasing time of immersion. The longer the colorant is in contact with the surface of restoration, the greater the discoloration observed. This result is in agreement with that of Özdaş et al. [16] who observed that all materials showed significant color change after all periods of time (*p* < 0.01) of immersion in different beverages.

Before and 24 h, 3 days, and 7 days after immersion, the pH of Yemeni coffee was 6.4, 5.9, 5.7, and 5.4 while that of red tea was 6.6, 6.1, 5.7, and 5.8, respectively. This is in agreement with the results of Özdaş et al. [16] who showed that Nescafe Classic coffee pH was 5.4 and that of Lipton black tea (Unilever, İstanbul, Turkey) was 6; both beverages were acidic, but Yemeni coffee was less acidic than was Nescafe, and red tea was less acidic than was black tea. Their study did not measure the pH after immersion; they examined the relationship between initial pH and discoloration.

Before immersion, Dilsi cola showed the lowest pH among all beverage immersion media, and the acidity increased with increasing immersion time. The negative effect of lower pH was seen in the wear of the composite materials and erosion of the polymer. The acidity in the beverage caused roughness on the surface, which helped accelerate discoloration. Dilsi cola had the most acidity but the lowest ∆E because Dilsi cola contains a weak colorant component, caramel; even though the surface was rough, the weak colorant led to low discoloration.

This result is in agreement with those of Gupta et al. [32] who showed that all the beverages used in their study were acidic, with Coca-Cola being the most acidic (pH = 1.57), followed by lemonade (pH = 2.32). Cola gains its color through the addition of caramel color, and red wine takes its color mainly from grapes. The stronger the colorant, the greater the discoloration.

The effects of Dilsi cola pH could be attributed to the susceptibility of the composite resin to chemical erosion of the resin matrix due to the low pH; this results in the hydrolytic breakdown of the filler particles and chemical degradation of the silane coupling agent, which can further be responsible for their discoloration [38]. Furthermore, low pH values of prospect colorant immersion media soften the resin matrix, and chemical erosion may occur. This degradation may cause more water absorption and attendant discoloration [39].

In this study, the pH of Dilsi cola before and 7 days after immersion was 3.1 and 2.8, respectively; it was the most acidic among all immersion media. The pH of water was 7.1 (neutral). Choi et al. [30] also showed that the beverage with the most acidic pH was cola, whereas water (control group) was neutral.

Discoloration was not associated with the pH value in this study. Qat was the highest discoloring agent with a 5.1 pH; although Dilsi cola was more acidic than was qat, it produced lesser discoloration. This result is in agreement with the results of Catelan et al. [40] who concluded that orange juice (pH 3.39) caused more color change than did cola soft drink, despite the lower pH of cola (2.36). This was attributed to different main acids rather than the pH of the staining solution and type of pigment.

This study had some limitations. First, the specimens had flat surfaces. In clinical settings, various anatomical features, including grooves and pits, may be present, and this may complicate the polishing procedure, and provisional restorations will have an irregular shape with convex and concave surfaces. This limitation could affect the results because it does not mimic reality; different anatomical features show different degrees of discoloration on the same tooth, unlike the flat surface of our samples, which had the same degree of discoloration on the entire surface.

Second, the concentration of solutions used as daily beverages is highly variable depending on the type of cuisine and individual preference. Therefore, the concentrations used in this study provide only a general estimation. Third, no artificial saliva was incorporated to mimic the oral environment in this study. Other factors could also influence the degree of total color change, including thermocycling processes and abrasion. Fourth, no comparisons were made between Yemeni coffee and other universal types since more precision would be required, such as the type of coffee, degree of roasting, whether the beans were processed or natural, the concentration of coffee in each cup, addition of sugar, as well as the difficulty of obtaining all types of Yemeni and international coffee.

## Conclusions

The nanohybrid composite was more color-stable than was the microhybrid composite. In composite resins, a smaller filler particle size increases the resistance to discoloration; therefore, the microhybrid composite (Te-Econom plus) was more susceptible to discoloration than was the nanohybrid composite (Tetric N-cream).

Qat caused greater discoloration of both materials than did other staining solutions and resulted in the most undesirable discoloration. Dilsi cola caused abrasion of the composite specimen, and this could facilitate discoloration. Time plays an important role in discoloration, as increasing time increases the ability of staining agents to cause discoloration (with the indirect role of pH).

## Data Availability

The datasets used and/or analysed during the current study are available from the corresponding author on reasonable request.
